# Eukaryotic translation initiation factor 3 subunit C is associated with acquired resistance to erlotinib in non-small cell lung cancer

**DOI:** 10.18632/oncotarget.26494

**Published:** 2018-12-25

**Authors:** Takuya Shintani, Kazuma Higashisaka, Makiko Maeda, Masaya Hamada, Ryosuke Tsuji, Koudai Kurihara, Yuri Kashiwagi, Atsuhiro Sato, Masanori Obana, Ayaha Yamamoto, Keisuke Kawasaki, Ying Lin, Takashi Kijima, Yuhei Kinehara, Yoshihiro Miwa, Shinichiro Maeda, Eiichi Morii, Atsushi Kumanogoh, Yasuo Tsutsumi, Izumi Nagatomo, Yasushi Fujio

**Affiliations:** ^1^ Graduate School of Pharmaceutical Sciences, Osaka University, Suita, Japan; ^2^ Department of Pharmacy, Osaka University Hospital, Suita, Japan; ^3^ Department of Legal Medicine, Osaka University Graduate School of Medicine, Suita, Japan; ^4^ Department of Pathology, Osaka University Graduate School of Medicine, Suita, Japan; ^5^ Division of Respiratory Medicine, Department of Internal Medicine, Hyogo College of Medicine, Nishinomiya, Japan; ^6^ Department of Respiratory Medicine and Clinical Immunology, Graduate School of Medicine, Osaka University, Suita, Japan; ^7^ Integrated Frontier Research for Medical Science Division, Institute for Open and Transdisciplinary Research Initiatives, Osaka University, Suita, Japan; ^8^ The Center for Advanced Medical Engineering and Informatics, Osaka University, Suita, Japan

**Keywords:** eukaryotic translation initiation factor 3 subunit C (eIF3c), non-small cell lung cancer (NSCLC), epidermal growth factor receptor (EGFR), EGFR-TKI resistance, autophagy

## Abstract

The acquisition of resistance to EGFR tyrosine kinase inhibitors (EGFR-TKIs) is one of the major problems in the pharmacotherapy against non-small cell lung cancers; however, molecular mechanisms remain to be fully elucidated. Here, using a newly-established erlotinib-resistant cell line, PC9/ER, from PC9 lung cancer cells, we demonstrated that the expression of translation-related molecules, including eukaryotic translation initiation factor 3 subunit C (eIF3c), was upregulated in PC9/ER cells by proteome analyses. Immunoblot analyses confirmed that eIF3c protein increased in PC9/ER cells, compared with PC9 cells. Importantly, the knockdown of eIF3c with its siRNAs enhanced the drug sensitivity in PC9/ER cells. Mechanistically, we found that LC3B-II was upregulated in PC9/ER cells, while downregulated by the knockdown of eIF3c. Consistently, the overexpression of eIF3c increased the number of autophagosomes, proposing the causality between eIF3c expression and autophagy. Moreover, chloroquine, an autophagy inhibitor, restored the sensitivity to erlotinib. Finally, immunohistochemical analyses of biopsy samples showed that the frequency of eIF3c-positive cases was higher in the patients with EGFR-TKI resistance than those prior to EGFR-TKI treatment. Moreover, the eIF3c-positive cases exhibited poor prognosis in EGFR-TKI treatment. Collectively, the upregulation of eIF3c could impair the sensitivity to EGFR-TKI as a novel mechanism of the drug resistance.

## INTRODUCTION

In spite of the recent advances in molecular targeted therapy, lung cancer remains the leading cause of cancer-related death worldwide [1]. Epidermal growth factor receptor (*EGFR*) is the main oncogenic driver in non-small cell lung cancer (NSCLC). Previous retrospective analyses have reported EGFR over-expression in 62% of NSCLC cases, and its expression is correlated with a poor prognosis [2]. The phosphorylation of EGFR initiates downstream pathways, such as Ras/extracellular signal-regulated kinase 1 and 2 (ERK1/2), phosphatidylinositol 3-kinase/Akt/mammalian target of rapamycin (mTOR), and Janus kinase 2/signal transducer and activator of transcription 3, leading to carcinogenesis by promoting cell proliferation, survival, invasion and metastasis [3]. In NSCLC patients, somatic mutations in *EGFR* gene, most commonly deletions in exon 19 (delE746-A750) or substitution of arginine for leucine (L858R) in exon 21, are observed in ∼10% of cases in North America and Western Europe, and ∼30–50% in East Asian descent [4–6]. EGFR-tyrosine kinase inhibitors (EGFR-TKIs), such as gefitinib [7, 8], erlotinib [9], and afatinib [10], display significant efficacy against *EGFR*-mutated NSCLC. However, lung tumors inevitably acquire resistance to these EGFR-TKIs around 12 months [11].

A secondary mutation (T790M), substitution of threonine for methionine in exon 20 of the *EGFR* gene, is the most common mechanism of EGFR-TKI resistance and accounts for 50% of EGFR-TKI resistant cases [12, 13]. Though osimertinib has been clinically approved for patients with lung tumors harboring *EGFR* T790M [14], the acquired resistance is unavoidable for osimertinib [15]. Indeed, *EGFR* T790M-independent mechanisms, such as *MET* [16, 17] or *HER2* [18] gene amplification, and AXL upregulation [19] have been reported so far. However, the intrinsic mechanisms of acquired resistance remain unclear in up to 40% of lung cancer patients [20]. Interestingly, recent studies demonstrated that the enhancement of autophagy causes EGFR-TKI resistance [21–23]. Autophagy is an intracellular catabolic process that maintains cellular energetic balance through the degradation of proteins and organelles in lysosomes [24]. Though environmental stimuli including chemotherapeutic agents induce autophagy [25], the mechanisms how EGFR-TKI resistant cells exhibit the enhanced autophagy are still unknown.

Translation, an essential process for maintenance of cellular functions, is mainly regulated at the initiation stage, which begins with the recruitment of the 40S ribosome subunit by eukaryotic translation initiation factors (eIFs) [26]. The eIF3 stabilizes the binding of the 40S subunit to the mRNA, contributing to the recognition of the starting AUG codon [27]. Human eIF3 complex is the largest eIFs protein complex composed of 13 subunits from eIF3a to 3m. The functional core of human eIF3 is composed of six subunits (eIF3a, 3b, 3c, 3e, 3f, and 3h) of which only eIF3a, 3b, and 3c are conserved in all eukaryotes and represent core units to which most of the other subunits bind [28, 29]. Recently, it has been reported that eIF3 selectively binds to mRNA related to cell proliferation and controls their translation [30]. Importantly, eIF3c is essential for cell proliferation in various human tumors [31–37]. However, it remains to be addressed whether eIF3c is involved in the resistance to anti-cancer drugs.

In this study, we newly established NSCLC cell lines with acquired resistance to erlotinib and compared the proteome between erlotinib-sensitive and resistant NSCLC cell lines. Interestingly, it was revealed that eIF3c was related to EGFR-TKI resistance and autophagy induction in cultured NSCLC cells. Moreover, the expression level of eIF3c is closely associated with the prognosis of NSCLC patients, who were treated with EGFR-TKIs. This is the first demonstration that eIF3c could be involved in the acquisition of EGFR-TKI resistance in NSCLC.

## RESULTS

### Establishment of cell line acquiring resistance to erlotinib

Initially, we analyzed the sensitivity of PC9 and PC9/ER cells to EGFR-TKIs (Supplementary Figure 1A). Cells were cultured with various concentrations of erlotinib or osimertinib, and then cell viability assay was performed. IC_50_ values of erlotinib and osimertinib were estimated at >10 μM and 3.05 μM in PC9/ER cells, respectively, while 7.64 nM and 9.19 nM in PC9 cells, indicating that PC9/ER cells exhibited strong resistance to erlotinib as well as to osimertinib. The resistance to erlotinib was maintained in PC9/ER cells after cells were cultured in the absence of erlotinib for 4 weeks (Supplementary Figure 1B). To examine whether gene mutations are related to EGFR-TKI resistance in PC9/ER cells, we performed the sequencing analysis of *EGFR* exons 18 to 21, which cover the tyrosine kinase domain of the receptor. As a result, there was no difference between PC9 and PC9/ER cells. Moreover, T790M mutation was not observed in PC9/ER cells (Supplementary Figure 1C), indicating that PC9/ER cells acquired EGFR-TKI resistance independently of T790M mutation.

### Proteomics analysis identified several translation-related proteins in erlotinib-resistant cell line

The proteomics analysis was performed to search for candidate proteins, which play key roles in the reduction of the sensitivity to EGFR-TKI. LC-MS/MS analysis showed 479 and 551 proteins were identified in PC9 and PC9/ER cells, respectively, among which 305 proteins were commonly detected in two cell lines. Then, we focused on 246 proteins that were identified exclusively in PC9/ER cells and performed pathway analysis. As a result, 12 pathways were involved in translation control among 36 pathways extracted as statistically significant (Table 1). GoSlim analysis also showed that ribosome was the most significant expression site of the proteins identified exclusively in PC9/ER cells (Supplementary Table 1), suggesting that the translation control function may alter in PC9/ER cells.

**Table 1:** List of pathways detected only in the EGFR-TKI resistant cell

Pathways	*p*-Value	Matches
Ribosome [hsa03010]	5.26E-11	17
Influenza Life Cycle [R-HSA-168255]	2.02E-10	17
Formation of a pool of free 40S subunits [R-HSA-72689]	2.20E-10	15
Peptide chain elongation [R-HSA-156902]	5.12E-10	14
Viral mRNA Translation [R-HSA-192823]	5.69E-10	14
Influenza Infection [R-HSA-168254]	6.89E-10	17
Influenza Viral RNA Transcription and Replication [R-HSA-168273]	8.24E-10	16
Selenocysteine synthesis [R-HSA-2408557]	1.05E-09	14
Eukaryotic Translation Termination [R-HSA-72764]	1.05E-09	14
Eukaryotic Translation Elongation [R-HSA-156842]	1.16E-09	14
SRP-dependent cotranslational protein targeting to membrane [R-HSA-1799339]	1.35E-09	15
Nonsense-Mediated Decay (NMD) [R-HSA-927802]	1.60E-09	15
Nonsense Mediated Decay (NMD) enhanced by the Exon Junction Complex (EJC) [R-HSA-975957]	1.60E-09	15
L13a-mediated translational silencing of Ceruloplasmin expression [R-HSA-156827]	1.88E-09	15
3′ -UTR-mediated translational regulation [R-HSA-157279]	1.88E-09	15
GTP hydrolysis and joining of the 60S ribosomal subunit [R-HSA-72706]	2.22E-09	15
Nonsense Mediated Decay (NMD) independent of the Exon Junction Complex (EJC) [R-HSA-975956]	2.51E-09	14
Selenoamino acid metabolism [R-HSA-2408522]	3.31E-09	15
Eukaryotic Translation Initiation [R-HSA-72613]	7.15E-09	15
Cap-dependent Translation Initiation [R-HSA-72737]	7.15E-09	15
Translation [R-HSA-72766]	3.96E-08	16
Major pathway of rRNA processing in the nucleolus [R-HSA-6791226]	2.99E-07	15
Gene Expression [R-HSA-74160]	7.08E-07	48
rRNA processing [R-HSA-72312]	1.01E-06	15
Spliceosome [hsa03040]	6.69E-06	13
Infectious disease [R-HSA-5663205]	7.62E-06	20
pre-mRNA splicing [R-HSA-72163]	1.77E-04	12
mRNA Splicing [R-HSA-72172]	2.87E-04	12
Amino acid and derivative metabolism [R-HSA-71291]	4.85E-04	17
Processing of Capped Intron-Containing Pre-mRNA [R-HSA-72203]	7.13E-04	13
Metabolism of proteins [R-HSA-392499]	0.004842	31
The citric acid (TCA) cycle and respiratory electron transport [R-HSA-1428517]	0.009636	11
Disease [R-HSA-1643685]	0.01285	24
RHO GTPases activate PKNs [R-HSA-5625740]	0.01575	7
Metabolism [R-HSA-1430728]	0.02835	43
Positive epigenetic regulation of rRNA expression [R-HSA-5250913]	0.0464	7

The pathway analysis was performed on protein detected only in the PC9/ER cells by the proteomics. There were 36 statistically significant pathways.

### eIF3c upregulated in erlotinib-resistant cell lines

**Figure 1:**
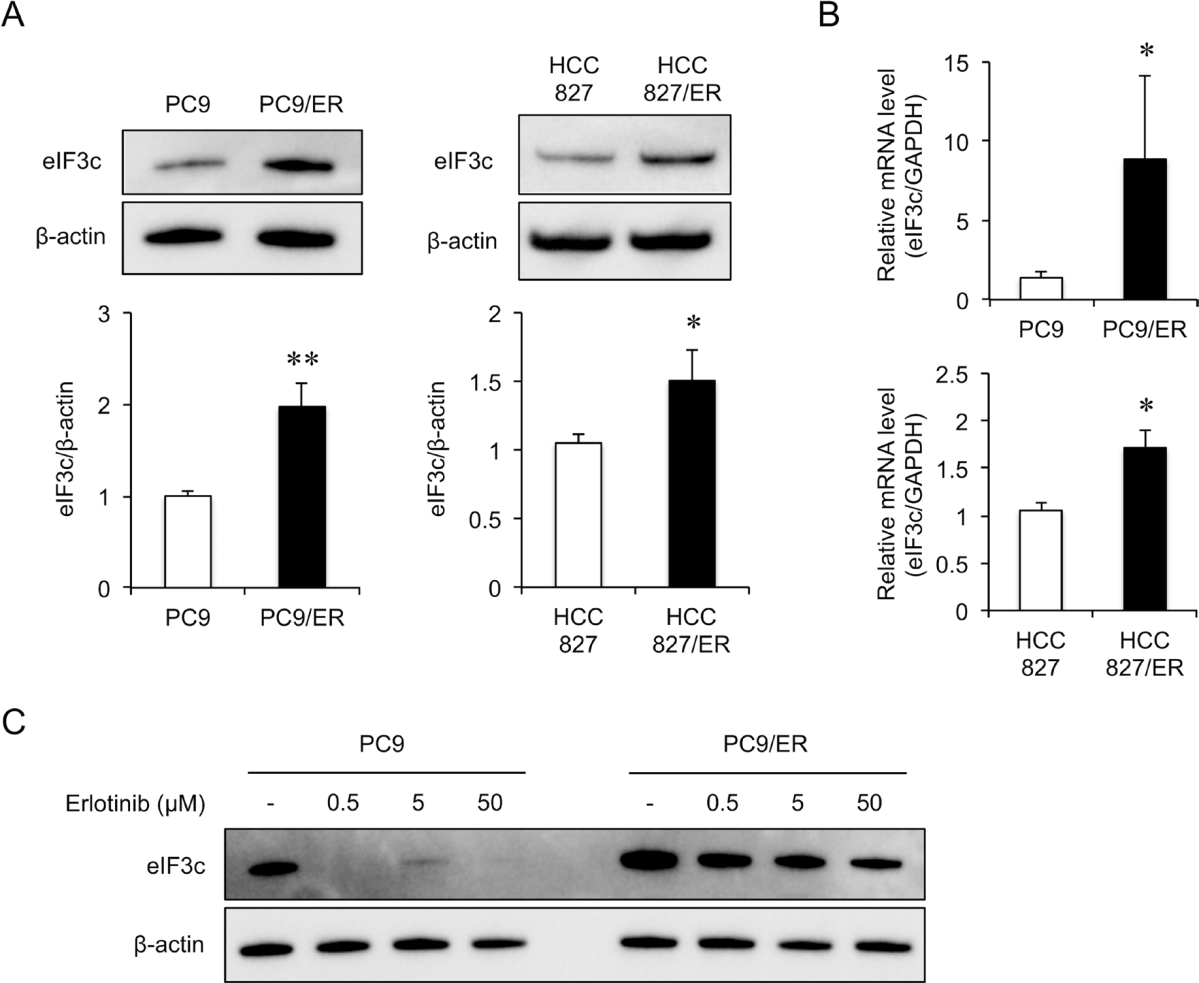
eIF3c increased in the EGFR-TKI resistant cell lines (**A**) Immunoblot analysis was performed in PC9, PC9/ER, HCC827 and HCC827/ER cells. Upper panels, representative images are shown. Lower panel, the amount of eIF3c protein was normalized with that of β-actin. (**B**) The expression of eIF3c transcripts was analyzed by RT-qPCR in PC9, PC9/ER, HCC827 and HCC827/ER cells, and normalized with that of GAPDH. (**C**) PC9 and PC9/ER cells were treated with the indicated concentrations of erlotinib for 24 h. The lysates were harvested, and the amount of eIF3c protein was analyzed by immunoblotting. Data are shown as the means ± SEM (*n* = 8 from 3 independent experiments). ^*^P < 0.05 and ^**^P < 0.01, compared with PC9 cells or HCC827 cells. *P*-values were determined by Student’s *t*-test.

Based on the results of the pathway analysis, we hypothesized that translation-related proteins are involved in erlotinib resistance and focused on eIF3c, which plays a critical role in the eukaryotic translation initiation (Supplementary Table 2). In PC9/ER cells, the protein level of eIF3c significantly increased compared to that in PC9 cells (Figure 1A), and the mRNA expression level also increased (Figure 1B). To examine the amount of eIF3c protein in the other EGFR-TKI resistant cell line, we established HCC827/ER cells from HCC827 cells. IC_50_ value of erlotinib was estimated at 7.20 μM in HCC827/ER cells, while 4.86 nM in HCC827 cells. We confirmed that T790M mutation was also undetected in HCC827/ER cells. The protein and mRNA level of eIF3c also significantly increased in HCC827/ER cells compared to those in HCC827 cells (Figure 1A, 1B). Next, we examined whether erlotinib affects the amount of eIF3c protein. Erlotinib drastically reduced eIF3c protein in PC9 cells, but eIF3c decreased much less prominently in PC9/ER cells (Figure 1C). These results implied that EGFR-TKI reduced eIF3c in erlotinib-sensitive cell lines but not in the resistant cells, proposing that eIF3c could contribute to erlotinib-resistance.

### Knockdown of eIF3c improved erlotinib sensitivity in PC9/ER cells

**Figure 2:**
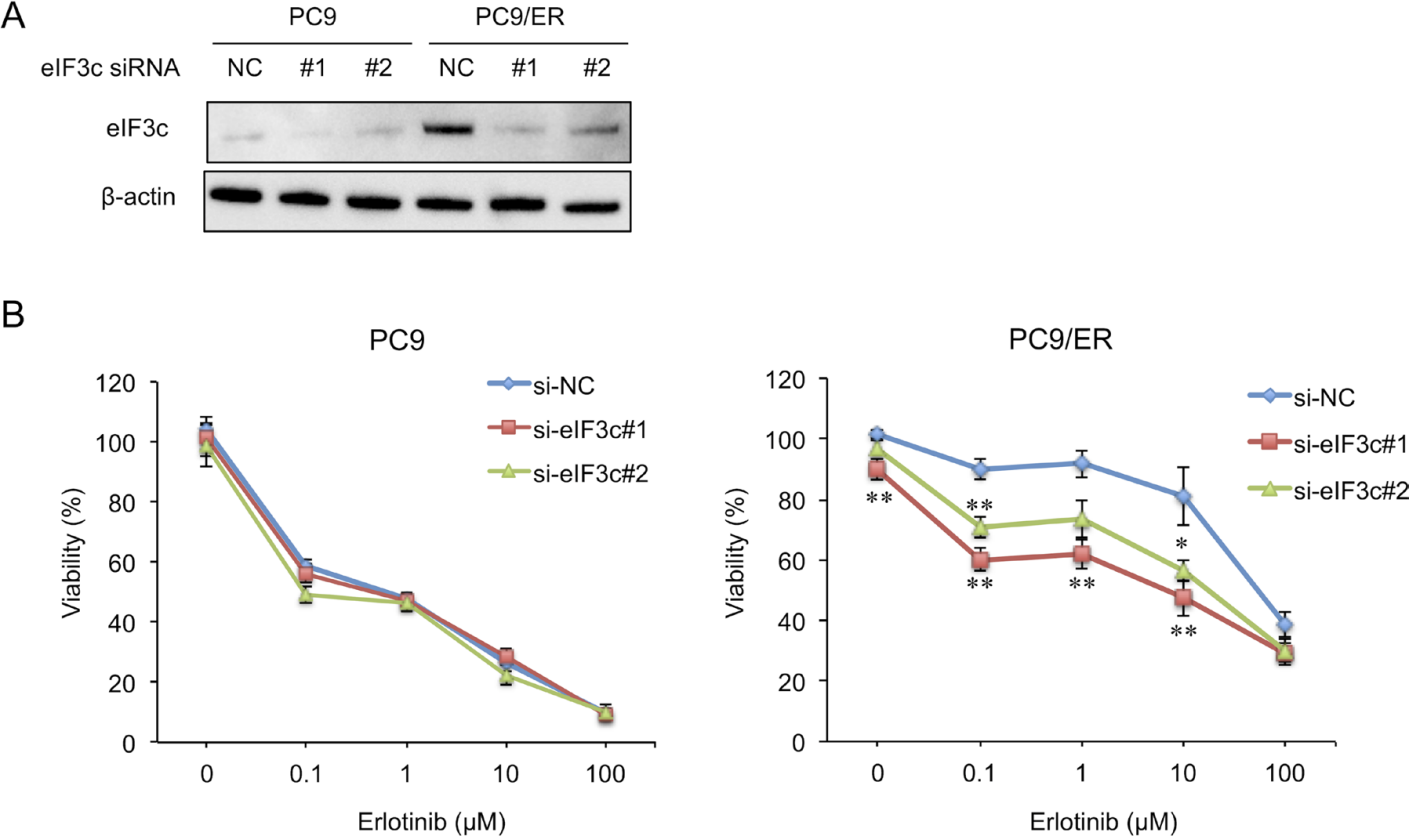
Knockdown of eIF3c expression enhanced erlotinib sensitivity in the EGFR-TKI resistant cell (**A**) PC9 and PC9/ER cells were transfected with siRNAs for eIF3c (#1 and #2) or with negative control siRNA (NC). After 72 h, the amount of eIF3c protein was analyzed by immunoblotting. (**B**) PC9 and PC9/ER cells were transfected with the indicated siRNAs and treated with the indicated concentrations of erlotinib for 72 h. Cell viability was measured by MTT assay. Data are shown as the means ± SEM (*n* = 12 from 4 independent experiments). ^*^P < 0.05 and ^**^P < 0.01, compared with si-NC. *P*-values were determined by ANOVA with Dunnett test in each concentration of erlotinib.

In order to examine whether eIF3c participates in EGFR-TKI resistance, eIF3c siRNA was transfected into PC9 and PC9/ER cells. Two kinds of eIF3c siRNAs, designated as #1 and #2, decreased the amount of eIF3c protein in both cells (Figure 2A). Importantly, the knockdown of eIF3c expression enhanced sensitivity to erlotinib in PC9/ER cells but not in PC9 cells (Figure 2B). Of note, the knockdown of eIF3c expression did not completely recover the sensitivity to erlotinib, possibly due to the incomplete inhibition of eIF3c expression. At the same time, we should notice the possibility that other pathways might be involved in the acquisition of EGFR-TKI resistance. Nonetheless, these results suggest that the upregulation of eIF3c at least partially contributed to EGFR-TKI resistance.

### The enhanced eIF3c augmented the autophagic activity

**Figure 3:**
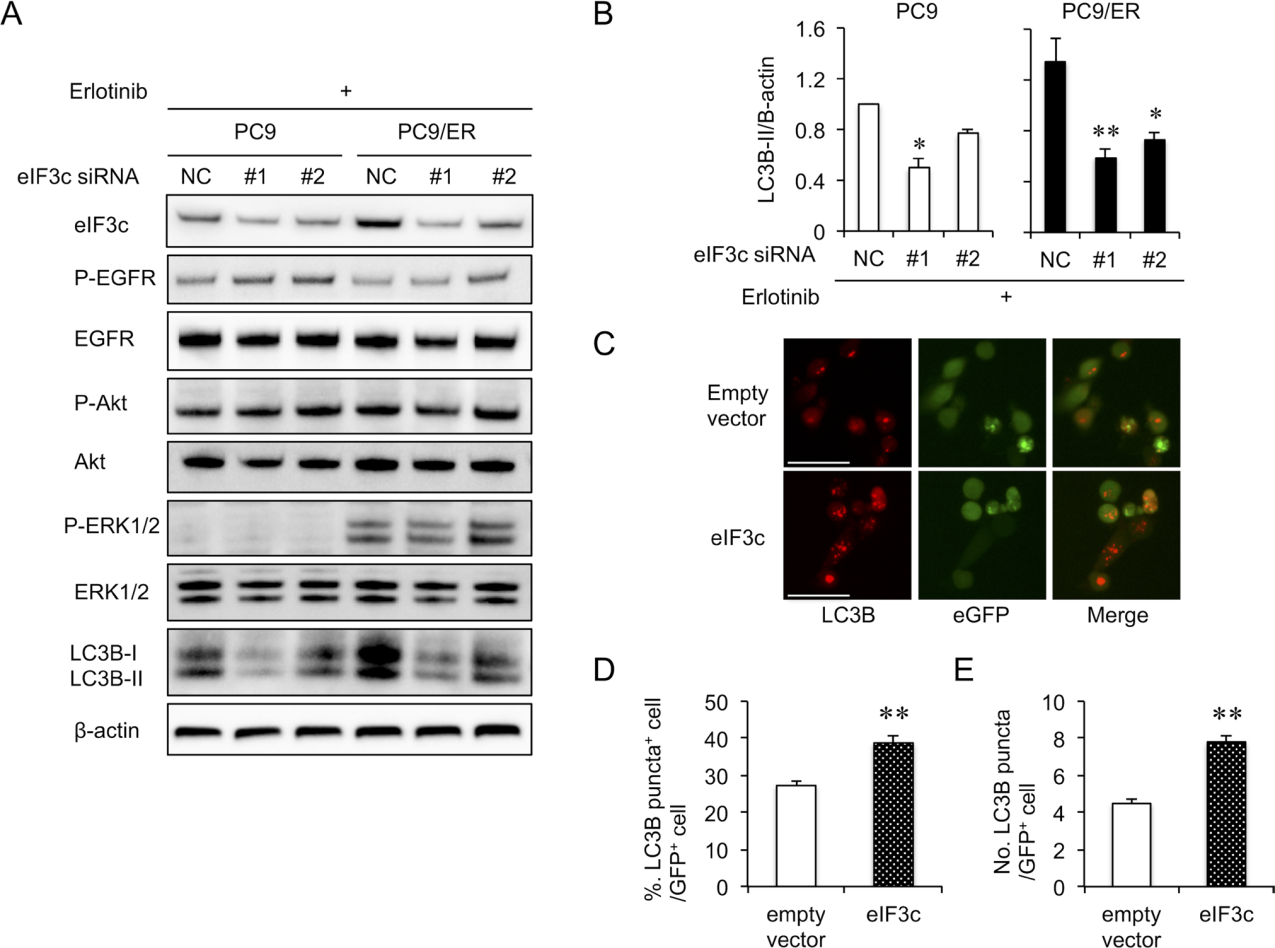
Autophagy activity was dependent on eIF3c expression level (**A**) PC9 and PC9/ER cells were transfected with siRNAs for eIF3c (#1 and #2) or with negative control siRNA (NC). Twenty-four hours later, cells were treated with erlotinib (5 μM) for 24 h. The signaling pathways related to EGFR-TKI resistance were analyzed. (**B**) The amount of LC3B-II protein was quantified and normalized with that of β-actin. Data are shown as the means ± SEM (*n* = 4 from 3 independent experiments). ^*^P < 0.05 and ^**^P < 0.01, compared with NC of each cell lines. *P*-values were determined by ANOVA with Dunnett test. (**C**–**E**) PC9 cells were transfected with the plasmid vectors expressing eIF3c, LC3B-RFP and eGFP, a transfection marker (eIF3c or empty vector : LC3B-RFP : eGFP = 3 : 2 : 1). Instead of the eIF3c vector, the empty vector was used as a negative control. Thirty hours after transfection, autophagosome formation was estimated as LC3B-RFP puncta. The experiments were repeated 3 times with similar results. (C) Representative images are shown. Scale bars = 50 μm. (D) The percentage of cells with more than two LC3B-RFP puncta, designated as puncta positive cell, was estimated in more than 500 eGFP positive cells. (E) The number of LC3B-RFP puncta was counted in more than 50 eGFP positive cells. Data are mean ± SEM. ^**^P < 0.01, compared with empty vector. *P*-values were determined by Student’s *t*-test.

To elucidate the mechanism by which eIF3c causes EGFR-TKI resistance, we transfected control or eIF3c siRNAs in PC9 or PC9/ER cells and evaluated the survival signals in the presence of erlotinib (Figure 3A). There was no significant difference in phosphorylation of EGFR and Akt between PC9 and PC9/ER cells. P-ERK1/2 increased in PC9/ER cells, but the knockdown of eIF3c expression did not influence P-ERK1/2, suggesting that ERK1/2 is unlikely to be a downstream effector of eIF3c. Though P-Met increased in PC9/ER cells (Supplementary Figure 2A), the inhibition of Met did not influence EGFR-TKI resistance (Supplementary Figure 2B). Interestingly, the protein level of LC3B-II, an autophagy marker, increased in PC9/ER cells and significantly decreased by eIF3c knockdown (Figure 3B). We transfected eIF3c expressing plasmid with LC3B-RFP, the autophagosome marker, into PC9 cells using eGFP as a transfection marker (Figure 3C–3E). The overexpression of eIF3c protein significantly increased the frequency of the LC3B puncta-positive cells (Figure 3D). Moreover, the number of LC3B puncta in eGFP positive cells also increased (Figure 3E). These results indicate that the upregulation of eIF3c could enhance autophagic activities.

### The activation of autophagy contributed to EGFR-TKI resistance in erlotinib-resistant cell line

**Figure 4:**
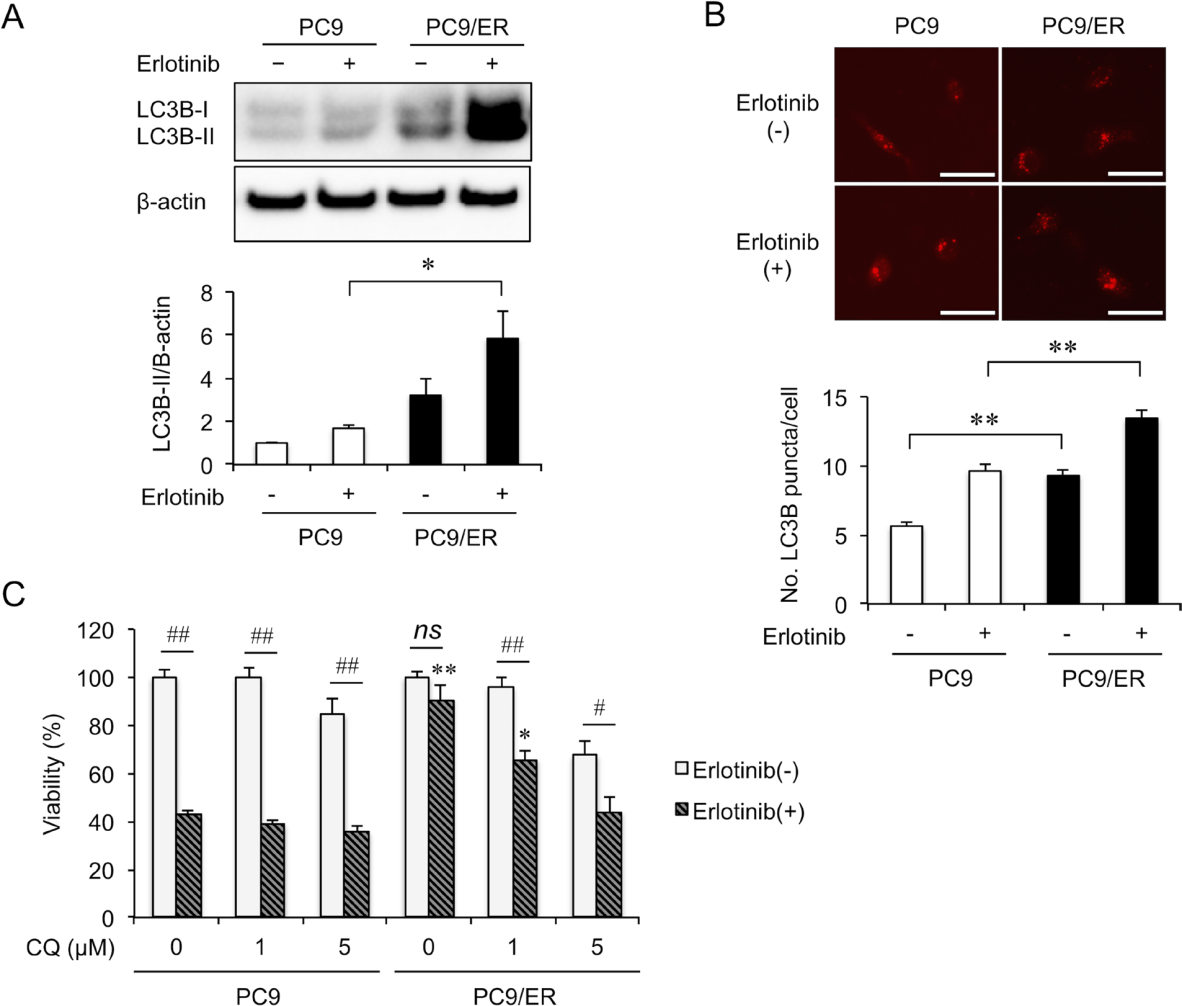
Autophagy is enhanced in the EGFR-TKI resistant cell (**A**) Cells were cultured in the absence of erlotinib for 4 days, followed by treatment with or without erlotinib (5 μM) for 3 h. LC3B-I/II was assessed by immunoblotting. Upper panels, representative images. Lower panel, the amount of LC3B-II protein was normalized with that of β-actin. Data are shown as mean ± SEM (*n* = 4 from 3 independent experiments). (**B**) Cells were transfected with the plasmid expressing LC3B-RFP and treated with or without erlotinib (5 μM) for 3 h. Upper panels, representative images. Lower panel, quantifications of LC3B-RFP puncta autophagosomes in PC9 and PC9/ER cells. Scale bars = 50 μm. Data are shown as mean ± SEM (*n* = 150 cells from 3 independent experiments). (A, B) ^*^P < 0.05, ^**^P < 0.01, ANOVA with Tukey-Kramer test. (**C**) PC9 and PC9/ER cells were treated with the indicated concentrations of chloroquine (CQ) with or without erlotinib (5 μM) for 72 h, and cell viability was measured by MTT assay. Data are shown as mean ± SEM (*n* = 16 from 3 independent experiments). ^#^*P* < 0.05 and ^##^*P* < 0.01. ^*^P < 0.05 and ^**^P < 0.01, compared with PC9 cells treated with the same concentration of CQ. *P*-values were determined by ANOVA with Tukey-Kramer test. The *ns* means no statistical difference.

To address the pathophysiological significance of autophagy in the acquisition of erlotinib resistance, we estimated the amount of LC3B-II protein in PC9 and PC9/ER cells in the presence or absence of erlotinib (Figure 4A). Compared with PC9 cells, LC3B-II increased in PC9/ER cells even in the absence of erlotinib. Importantly, the incubation of the cells with erlotinib further increased LC3B-II in PC9/ER cells, but to a lesser extent in PC9 cells. Similarly, when PC9 and PC9/ER cells were transfected with LC3B-RFP, an increase in autophagosomes was observed in PC9/ER cells (Figure 4B). Furthermore, we examined the effect of chloroquine (CQ), an autophagy inhibitor, on the resistance of PC9/ER cells to erlotinib (Figure 4C). Chloroquine partially decreased cell viability in PC9 and PC9/ER cells on its own. Importantly, chloroquine (5 μM) completely abrogated the resistance of PC9/ER cells to erlotinib. Interestingly, serum starvation-mediated induction of LC3B-II was more remarkably in PC9/ER cells than in PC9 cells (Supplementary Figure 3), confirming the importance of eIF3c in autophagy.

### eIF3c was clinically associated with EGFR-TKI resistance

**Figure 5:**
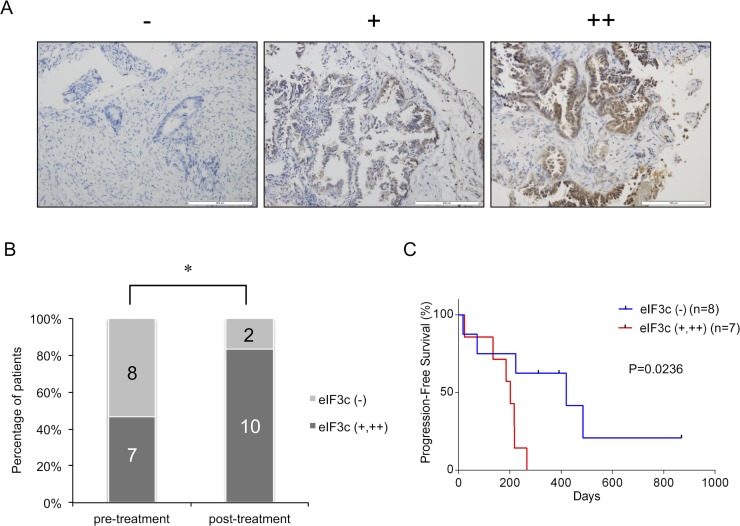
eIF3c was associated with the response to EGFR-TKI in patients harboring *EGFR*-active mutations (**A**) Biopsy samples from patients with non-small cell lung cancers (NSCLC) were immunostained with the anti-eIF3c antibody. According to the intensity of eIF3c staining, samples were classified into 3 groups, negative (–), weakly positive (+), and highly positive (++) by the researchers blinded to the clinical profiles of the patients. Representative images are shown. Scale bar = 200 μm. (**B**) The percentage of patients classified into positive (+ and ++) or negative (–) group in the pre-EGFR-TKI treatment group was compared with those in the post-EGFR-TKI treatment group who exhibited EGFR-TKI resistance. The number of patients for each condition was shown. ^*^P < 0.05, Fisher’s exact test. (**C**) Fifteen patients in the pre-EGFR-TKI treatment group were treated with EGFR-TKIs. Progression-free survival curves were shown. *P*-values were determined by the Kaplan–Meier method.

To make clear the clinical importance of eIF3c in the resistance to EGFR-TKI, we performed eIF3c immunohistochemistry analysis on 27 lung tumor biopsy samples from *EGFR*-sensitizing mutant NSCLC patients. Among the 27 samples, 15 samples were biopsy specimens before EGFR-TKI treatment and 12 cases were re-biopsy specimens from the patients with EGFR-TKI resistance after the treatment. According to the intensity of the staining, samples were classified into three levels: negative: –; weakly positive: +; and highly positive: ++ (Figure 5A). The positive rate of eIF3c (+ or ++) in the post-treatment (10/12 cases; 83.3%) was significantly higher than that in the pre-treatment (7/15; 46.7%) (*P* = 0.0473) (Figure 5B). In the post-treatment groups, T790M mutation was detected from 7 cases (58.3%), and all of them were eIF3c positive (+ and ++). Next, in order to investigate the relationship between the expression of eIF3c and prognosis, we performed Kaplan–Meier analysis on survival after starting EGFR-TKI therapy in the EGFR-TKI pre-treatment group (Figure 5C). The median progression-free survival (PFS) was 420 days in eIF3c negative (–) patients, while 202 days in eIF3c positive (+ and ++) patients (hazard ratio [HR], 0.315; 95% confidence interval [CI], 0.063–0.730; *P* = 0.0236). These results suggest that eIF3c expression could be positively correlated with EGFR-TKI resistance acquisition.

## DISCUSSION

In this study, we identified eIF3c as a novel molecule involved in EGFR-TKI resistance acquisition by newly establishing EGFR-TKI resistant cells, PC9/ER cells. The proteomics analysis revealed the translation-related proteins were abundantly identified in PC9/ER cells, compared with PC9 cells. Among them, we found that eIF3c increased in PC9/ER cells. The knockdown of eIF3c expression with siRNA attenuated EGFR-TKI resistance in PC9/ER cells. Interestingly, LC3B-II was upregulated in PC9/ER cells, which was enhanced by erlotinib, with the increased autophagic activity. Importantly, the knockdown of eIF3c expression suppressed autophagic activity, while overexpression of eIF3c reinforced autophagy. Moreover, the inhibition of autophagy with chloroquine impaired EGFR-TKI resistance. Finally, using the biopsy samples, we demonstrated that eIF3c was clinically associated with EGFR-TKI resistance and could be a biomarker for the prognosis after EGFR-TKI treatment in NSCLC patients.

To address the mechanism of EGFR-TKI resistance acquisition of PC9/ER cells, we performed proteomics analyses. As a result, it was proposed that translation control factors could modulate the responsiveness to EGFR-TKI. In this study, we focused on eIF3c, because overexpression of eIF3c enhances the translation function and malignancy in cancer cells [38]. eIF3c is known as one of the core subunits of eIF3 that is responsible for AUG recognition [39]. Interestingly, eIF3 binds directly to mTOR and activates cap-dependent translation via eIF4 and ribosomal protein S6 kinase [40]. Previously, it was reported that mTOR [41] and eIF4 [42, 43] are related to EGFR-TKI resistance. And, here, we demonstrated that eIF3c is involved in EGFR-TKI resistance for the first time.

It has been identified that a number of signaling pathways are responsible for EGFR-TKI resistance, including T790M mutation of *EGFR* [12, 13], activation of Met [16, 17], Akt [44], and ERK [45, 46]. Though T790M is one of the most well-known mutations responsible for the resistance, PC9/ER cells did not have T790M mutation. Among various other candidate pathways, we found that the expression of LC3B-II, an autophagy marker, was enhanced in PC9/ER cells in an eIF3c-dependent manner. Importantly, erlotinib treatment induced autophagy, and erlotinib-induced autophagy was reinforced in PC9/ER cells. Furthermore, we found that suppression of eIF3c decreased erlotinib-induced autophagy and that the overexpression of eIF3c enhanced autophagy, indicating that eIF3c is a major regulator of erlotinib-induced autophagy. Importantly, the inhibition of autophagy by chloroquine improved the sensitivity to erlotinib in PC9/ER cells. These results indicate that EGFR-TKI resistance of PC9/ER cells is dependent on autophagy. Consistent with our findings, it was reported that the inhibition of autophagy abrogates erlotinib resistance in NSCLC cells [47–49]; however, autophagy plays important roles in various kinds of biological functions [50], raising the concern that pharmacological inhibition might lead to serious adverse drug reactions. In this context, the development of eIF3 inhibitors could be a promising strategy against the resistance to EGFR-TKIs.

It remains to be clarified how eIF3c modulates autophagy; however, it should be noted that eIF3c enhanced autophagy in response to erlotinib. As EGF activates glutamine uptake [51], the promoted protein synthesis by eIF3c overexpression might result in a severe amino acid deficiency in the presence of EGFR-TKI. Importantly, amino acid deficiency-induced autophagy exhibits protective functions [52]. Consistent with our results, other eIFs, including eIF2α [53] and eIF5A [54], enhance autophagy, though it is unknown whether eIF2- or eIF5-mediated autophagy contributes to the drug responses. Collectively, we propose that the activation of translation through eIF3c promotes autophagy as a novel EGFR-TKI resistance mechanism.

Finally, we addressed the clinical significance of our findings. The frequency of eIF3c-positive samples was higher in biopsy specimens from the patients’ refractory to EGFR-TKI therapy than in those from the patients prior to the treatment with EGFR-TKI. Moreover, eIF3c-positive patients exhibited poor prognosis, suggesting that eIF3c could be a potential biomarker of responsiveness to the EGFR-TKI. However, we have noticed the limitations of these clinical observations. First, the sample number is still small, mainly because this study was performed at a single center. In addition, there were cases in which re-biopsy could not be performed depending on the tumor lesion. Moreover, in order to clarify the relation between the expression of eIF3c and the resistance to EGFR-TKI, it would be better to collect the biopsy samples from the same patients sequentially, though it will extremely limit the sample size. Finally, the prognosis was estimated as a retrospective study. Nonetheless, the clinical findings presented here would provide the rationale for the clinical researches to support the evidence that eIF3c can contribute to precision medicine as a biomarker for NSCLC patients.

In conclusion, the increased eIF3c expression enhances autophagy, which induces the resistance to EGFR-TKI in NSCLC, though further studies would be required to make clear how eIF3c modulates autophagy. Moreover, the patients with increased eIF3c expression in biopsy samples showed a poor prognosis. The inhibition of eIF3c could be a new therapeutic strategy for overcoming EGFR-TKI resistance in NSCLC patients.

## MATERIALS AND METHODS

### The human NSCLC cell lines and cell culture

PC9 cells (*EGFR* exon 19 deletion. E746-A750) were purchased from the Riken BioResource Center (Tsukuba, Japan) in July 2015. HCC827 cells (*EGFR* exon 19 deletion. E746-A750) were kindly gifted by Dr. Bruce E Johnson (Dana-Farber Cancer Institute, Boston, MA, USA). Cell authentication was last performed by STR profiling in December 2016. Both cell lines were cultured in RPMI 1640 medium (nacalai tesque, Kyoto, Japan) supplemented with 10% fetal bovine serum (FBS) (Dainippon Pharmaceutical, Osaka, Japan) and grown in a humidified incubator with 5% CO_2_ at 37° C.

### Establishment of EGFR-TKI resistant NSCLC cell lines

The PC9 and HCC827 cells were exposed to increasing concentrations of erlotinib in order to generate erlotinib-resistant cell lines (PC9/ER and HCC827/ER). PC9/ER and HCC827/ER cells were exposed to erlotinib with a stepwise escalation from 10 nM (PC9/ER) or 2.5 nM (HCC827/ER) to 5 μM over 6 months. PC9/ER and HCC827/ER cells were routinely cultured with medium containing 5 μM erlotinib.

### Reagents and antibodies

Erlotinib and osimertinib were purchased from Selleck Chemicals (Houston, TX, USA). Crizotinib was from Sigma-Aldrich (St. Louis, MO, USA). Chloroquine was obtained from Santa Cruz Biotechnology (Santa Cruz, CA, USA). The primary antibodies used for immunoblot analyses were the products of Cell Signaling Technology (Danvers, MA, USA); anti-eIF3c (#2068), EGFR (#4267), phospho-EGFR (Tyr1068) (#3777), Akt (#9272), phospho-Akt (Ser473) (#9271), ERK1/2 (#9102), phospho-ERK1/2 (Thr202/Tyr204) (#9101), Met (#8198), phospho-Met (Tyr1234/1235) (#3077), LC3B (#3868), and β-actin (#4970).

### Cell viability assay

To perform MTT assay, 5,000 to 6,000 cells per well were plated in 96-well sterile plastic plates and allowed to attach overnight; the cells were then exposed to different concentrations of erlotinib for 72 h, and MTT solution (0.5 mg/mL in medium) was added in each well. After incubation for 4 h at 37° C, the supernatant was removed and 100 μL isopropanol/0.04 N HCl was placed in each well to dissolve formazan for 15 min with gentle shaking at room temperature. Absorbance at 570 nm was determined on a microplate reader (SH-9000Lab; Hitachi High-Tech Science, Tokyo, Japan).

### Immunoblotting

Cells were washed in ice-cold phosphate-buffered saline (PBS) and lysed in cell extraction buffer (10 mM Tris HCl, pH 7.4, 1% Triton X-100, 100 mM sodium chloride, 0.1% SDS), both containing protease and phosphatase inhibitors (nacalai tesque). Protein concentrations were determined by Pierce BCA Protein Assay (Thermo Fisher Scientific, Rockford, IL, USA) according to the manufacturer’s protocol. Proteins (2.5–20 μg) were resolved on NuPAGE Novex 4–12% Bis-Tris Gel (Invitrogen, Grand Island, NY, USA) and then transferred onto PVDF membrane (Invitrogen). After blocking with TBS containing 0.1% Tween 20 and 1% non-fat dry skimmed milk for 1 h at room temperature, the membranes were incubated with primary antibodies overnight at 4° C, followed by the incubation with goat anti-rabbit HRP-conjugated secondary antibody (#7074, Cell Signaling Technology) in 1:2000 dilution for 1 h at room temperature. The immune reactive bands were visualized by Amersham ECL Prime Western Blotting Detecting Reagent (GE Healthcare, Buckinghamshire, England, UK) with a ChemiDoc Imaging System (Bio-Rad Laboratories, Hercules, CA, USA).

### Proteomics

Protein was collected from cell lines by PTS buffer (12 mM sodium deoxycholate, 12 mM N-Dodecanoyl sarcosinate, 50 mM Ammonium bicarbonate, Protease inhibitor cocktail) and purified by using 2-D Clean-Up Kit (GE Healthcare). After the sample was heated in 95° C for 5 min, 0.25 M DTT, 0.375 M IAA, and 50 mM Ammonium bicarbonate were added by steps to each sample with vortex, and then incubated in room temperature for 30 min. Trypsin was added into each protein sample and incubated at 37° C overnight. Then, samples were added 1% Trifluoroacetic Acid in ethyl acetate to each sample with vortex and centrifuged in 15,600 g for 3 min. To dry them up, Speedvac (TOMY SEIKO, Tokyo, Japan) was performed for 30 min and re-suspended by 2 M Urea and 1% TFA. After centrifuging in 20,000 g for 3 min, the supernatant was separated into 7 fractions by the tips balance adjustment. Dry up all fractionations by speedvac and re-suspended with 20% Paraformaldehyde and buffer contained with1% TFA, 2% ACN. The protein in samples was analyzed by LC-MS/MS (Thermo Fisher Scientific) and identified proteins by Mascot search with the databank of Swiss-Prot. The pathway analysis was performed to make clear the relation among the identified proteins using Targetmine (https://targetmine.mizuguchilab.org/).

### DNA sequencing

DNA was isolated from cell lines by Purelink genomic DNA mini kit (Thermo Fisher Scientific). EGFR tyrosine kinase domains (exon 18–21) in PC9 and PC9/ER cells were amplified by PCR and mutations were detected with Sanger sequencing. For detection of T790M in each cell lines, TaqMan Mutation Detection Assay (Thermo Fisher Scientific) was also performed.

*EGFR*-exon 18

Forward 5′-CAAATGAGCTGGCAAGTGCCGTGTC-3′

Reverse 5′-GAGTTTCCCAAACACTCAGTGAAAC-3′

*EGFR*-exon 19

Forward 5′-GCAATATCAGCC TTAGG TGCGGCTC-3′

Reverse 5′-CATAGAAAGTGAACATTTAGGATGTG-3′

*EGFR*-exon 20

Forward 5′-CCATGAGTACGTATTTTGAAACTC-3′

Reverse 5′-CATATCC CCATGGC AAACTCTTGC-3′

*EGFR*-exon 21

Forward 5′-CTAACGTTCGCCAG CCATAAGTCC-3′

Reverse 5′-GCTGCGAGCTCACCCAGAATGTCTGG-3′

### Quantitative real-time RT-PCR

Total RNA was extracted with TRIzol reagent (Invitrogen). For quantitative reverse transcription polymerase chain reaction (RT-PCR) analysis of mRNA expression, 0.5 μg of total RNA was reverse transcribed into cDNA in 10 μL of reaction system using a PrimeScript RT reagent Kit (Takara Bio, Kusatsu, Japan). Real-time PCR was conducted on the 7900HT Fast Real-Time PCR System (Applied Biosystems, Foster, CA, USA) using SYBR Premix Ex Taq (Takara Bio). The expression was normalized to glyceraldehyde-3-phosphate dehydrogenase (GAPDH) levels calculated by the 2^-ΔΔTc^ method.

eIF3c primer

Forward 5′-ACCAAGAGAGTTGTCCGCAGTG-3′

Reverse 5′-TCATGGCATTACGGATGGTCC-3′

GAPDH primer

Forward 5′-GCACCGTCAAGGCTGAGAAC-3′

Reverse 5′-TGGTGAAGACGCCAGTGGA-3′

### RNA interference

RNA interference was achieved by transfection of small interfering RNAs (siRNAs) against eIF3c (Silencer Select siRNAs, Invitrogen), si-eIF3c#1 (s58907) and si-eIF3c#2 (s225015), or control (Silencer Select Negative Control #1 siRNA, Invitrogen) using Lipofectamine RNAi-MAX (Invitrogen) according to the manufacturer’s instruction. Four hours after transfection, the transfection medium (Opti-MEM; Gibco) was replaced to RPMI 1640 medium with 10% FBS, and the cells were incubated for the indicated times. Knockdown efficiency was assessed by RT-PCR or immunoblotting.

### Plasmid transfection

The plasmid of eIF3c (OriGene Technologies, Rockville, MD, USA), LC3B-RFP (OriGene Technologies), and empty vector were transfected using FuGENE HD Transfection Reagent (Promega, Tokyo, Japan) according to the manufacturer’s instructions. Briefly, FuGENE HD Transfection Reagent (3 μL) and the plasmid (1 μg) were diluted in OPTI-MEM medium to 50 μL (in the absence of antibiotics or fungicides). The mixture was incubated for 15 min at room temperature. Finally, the transfection mixture was added to the culture dishes. After 30 h, the cells were processed for fluorescence microscopy.

### Patients and tissue samples

The tissue samples used in this study were paraffin-embedded blocks of tumor tissues archived at the Department of Pathology, Osaka University Graduate School of Medicine (Suita, Japan). Research protocols for this study were approved by the Ethics Committee at Osaka University Hospital (Approval number: 17252).

### Immunohistochemistry

Serial sections of 3 μm thickness were prepared from the paraffin-embedded tumor tissues. After deparaffinization, the sections were incubated with Dako Target Retrieval Solution, pH 9 (Dako, Glostrup, Denmark) at 125° C for 30 sec, 90° C for 10 min to retrieve antigen and then treated with peroxidase-blocking solution (Dako) for 5 min at room temperature to block endogenous peroxidase. The samples were incubated with the eIF3c antibody (Abcam, Cambridge, UK, #ab170841, dilution 1:50) for 30 min at room temperature. Then the samples were treated with the EnVision System (Dako). DAB+ (3–3′-Diaminobenzidine Tetrahydrochloride) Liquid (Dako) was used for color development. After nucleus staining with hematoxylin, the sections were examined under a light microscope. The intensity of the staining was scored as negative: –; weakly positive: +; and highly positive: ++, respectively. The scores were all from the visual estimation of a blinded pathologist.

### Statistics

Results were expressed as mean ± standard error of the mean (SEM). The two-tailed Student’s *t*-test was used for comparing the mean values between the two groups. One-way analysis of variance (ANOVA) and post-hoc analysis was applied to the comparison of three or more groups. Fisher’s exact test was used to compare categorical characteristics across groups. A survival curve was generated according to the Kaplan–Meier method, and statistical significance was calculated using the Log-rank test. A value of *P* < 0.05 was considered to be statistically significant. JMP pro version 13.0.0 (SAS, Tokyo, Japan) and GraphPad Prism, version 6.0.7, J (GraphPad Software, San Diego, CA, USA) software were used for statistical analysis.

## SUPPLEMENTARY MATERIALS FIGURES AND TABLES


