# Supplementary material for: Eukaryotic translation initiation factor 3 subunit C is associated with acquired resistance to erlotinib in non-small cell lung cancer
Source: Oncotarget. 2018 Dec 25;9(101):37520–33. doi: 10.18632/oncotarget.26494 (PMC6331022; doi:10.18632/oncotarget.26494)
Supplement: Supplementary file 1 [file oncotarget-09-37520-s001.pdf]

Eukaryotic translation initiation factor 3 subunit C is associated with acquired resistance to erlotinib in non-small cell lung cancer

SUPPLEMENTARY MATERIALS

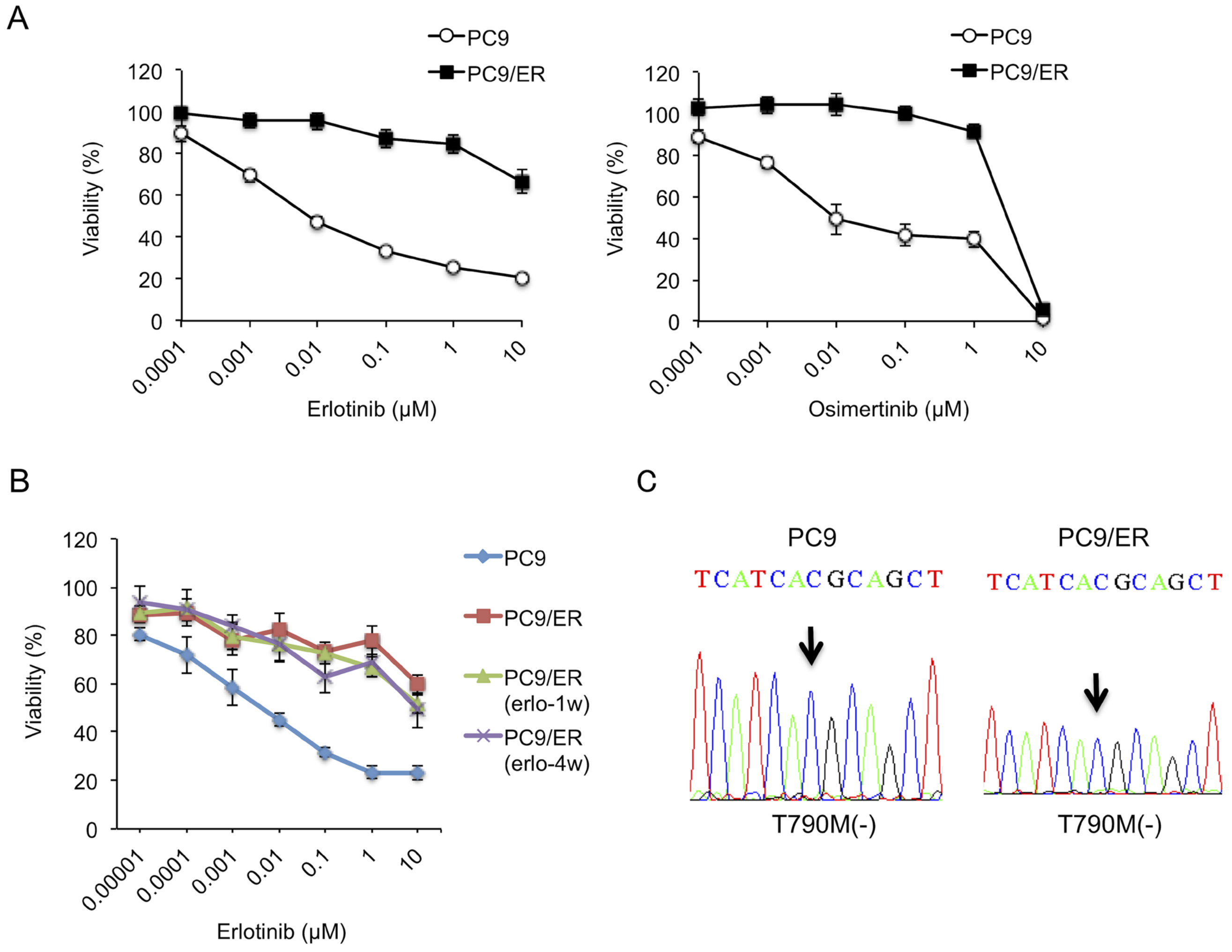

Supplementary Figure 1: PC9/ER cells acquired EGFR-TKI resistance. (**A**) PC9 and PC9/ER cells were treated with the indicated concentrations of erlotinib or osimertinib for 72 h, and cell viability was measured by MTT assay. Data are shown as the means ± SEM (n = 18 from 3 independent experiments). (**B**) PC9 and PC9/ER cells were treated with the indicated concentrations of erlotinib for 72 h, and cell viability was measured by MTT assay. To examine whether EGFR-TKI resistance was irreversible, PC9/ER cells were cultured in the absence of erlotinib (5 μM) for 1 week (erlo-1w) or 4 weeks (erlo-4w). The results are shown as the means ± SD (n = 6 from 1 experiment). (**C**) Sequences of *EGFR* exon 20 in PC9 and PC9/ER cells. Allows indicate *EGFR* T790M sites.

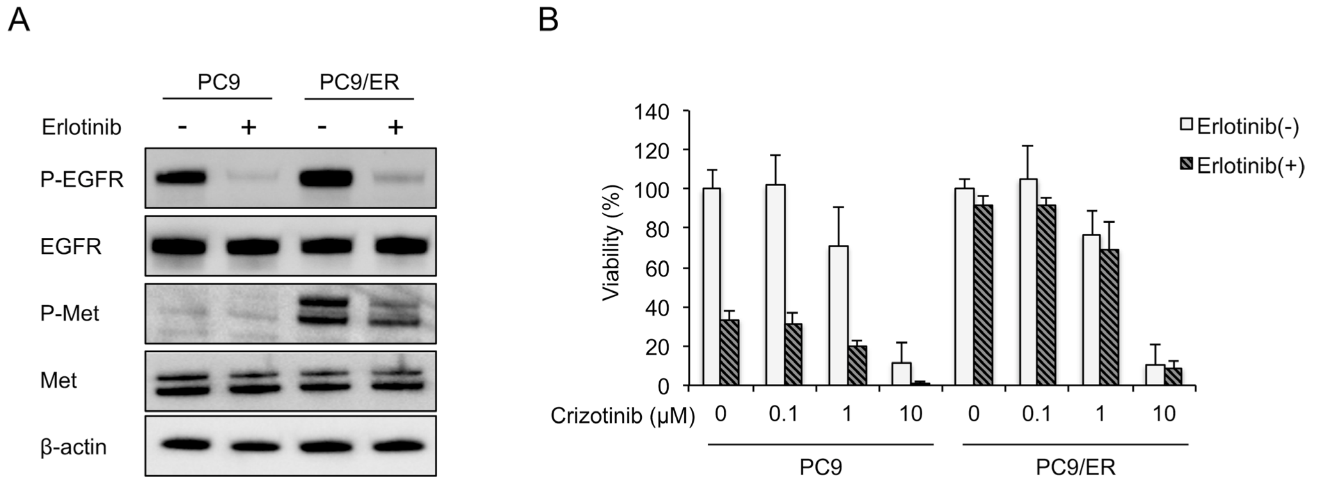

Supplementary Figure 2: The enhanced phosphorylation of Met in PC9/ER cells was not related with EGFR-TKI resistance. (**A**) Cells were cultured in the absence of erlotinib for 4 days and treated with or without erlotinib (5 μM) for 3 h. The phosphorylation status of EGFR (Tyr1068) and Met (Tyr1234/1235) were assessed by immunoblotting. (**B**) PC9 and PC9/ER cells were treated with the indicated concentrations of Met inhibitor (crizotinib) with or without erlotinib (5 μM) for 72 h, and cell viability was measured by MTT assay. Data are shown as the means ± SD (*n* = 21 from 4 independent experiments).

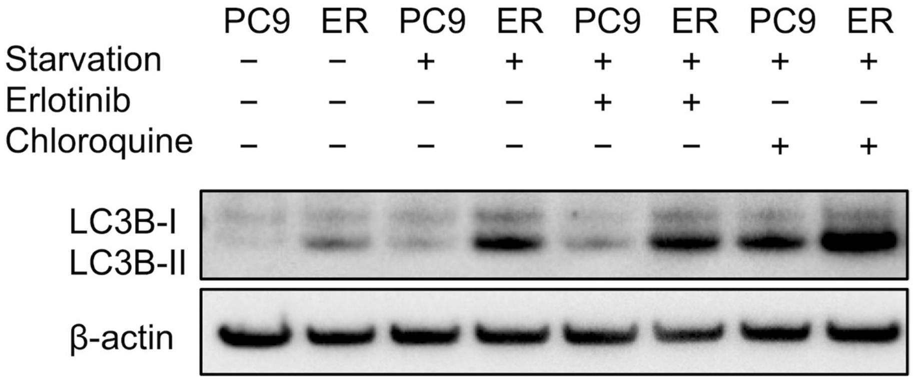

Supplementary Figure 3: Serum starvation-induced autophagy was enhanced in the EGFR-TKI resistant cell. PC9 and PC9/ER (ER) cells were cultured in serum-free medium (starvation), or serum-free medium plus erlotinib (5 μM) or chloroquine (5 μM) for 3 h. The amount of LC3B-I/II proteins was assessed by immunoblotting.

Supplementary Table 1: List of expression site of protein detected only in the EGFR-TKI resistant cell

GOSlim term	*p*-Value	Matches
ribosome [GO:0005840]	1.68E-12	19
organelle [GO:0043226]	3.77E-10	138
intracellular [GO:0005622]	2.70E-06	136
extracellular region [GO:0005576]	1.17E-05	66
mitochondrion [GO:0005739]	4.23E-05	28
cytoplasm [GO:0005737]	1.09E-04	109
nuclear chromosome [GO:0000228]	2.00E-04	14
chromosome [GO:0005694]	5.03E-04	17
nucleus [GO:0005634]	5.53E-04	75
nucleolus [GO:0005730]	0.003833	16
nucleoplasm [GO:0005654]	0.008034	43
lysosome [GO:0005764]	0.020015	10
cytosol [GO:0005829]	0.02961	42
protein complex [GO:0043234]	0.034612	44

The GoSlim analysis was performed on protein detected only in PC9/ER cells by the proteomics.

Supplementary Table 2: List of proteins included in the Eukaryotic translation initiation pathway

Protein primary accession	Protein name	Pathways identifier
P40429	60S ribosomal protein L13a	R-HSA-72613
P42766	60S ribosomal protein L35	R-HSA-72613
P46778	60S ribosomal protein L21	R-HSA-72613
P47914	60S ribosomal protein L29	R-HSA-72613
P61353	60S ribosomal protein L27	R-HSA-72613
P62241	40S ribosomal protein S8	R-HSA-72613
P62266	40S ribosomal protein S23	R-HSA-72613
P62269	40S ribosomal protein S18	R-HSA-72613
P62829	60S ribosomal protein L23	R-HSA-72613
P62841	40S ribosomal protein S15	R-HSA-72613
P62891	60S ribosomal protein L39	R-HSA-72613
P62987	Ubiquitin-60S ribosomal protein L40	R-HSA-72613
Q99613	Eukaryotic translation initiation factor 3 subunit C	R-HSA-72613
Q9UNX3	60S ribosomal protein L26-like 1	R-HSA-72613
Q9Y3U8	60S ribosomal protein L36	R-HSA-72613

The pathway analysis was performed on protein detected only in PC9/ER cells by the proteomics. The eukaryotic translation initiation pathway contained 15 proteins, including eIF3c.
